# First report of the bent seed gall nematode, *Anguina agrostis* (Steinbuch, 1799) Filipjev, 1936 from *Poa palustris* L. in Wyoming, USA

**DOI:** 10.21307/jofnem-2020-121

**Published:** 2020-11-30

**Authors:** Tatiana V. Roubtsova, Sergei A. Subbotin

**Affiliations:** 1 Department of Plant Pathology, University of California, One Shields Avenue, Davis, CA, 95616; 2 Plant Pest Diagnostic Center, California Department of Food and Agriculture, 3294 Meadowview Road, 95832, Sacramento, CA; 3 Center of Parasitology of A.N. Severtsov Institute of Ecology and Evolution of the Russian Academy of Sciences, Leninskii Prospect 33, 117071, Moscow, Russia

**Keywords:** Anguina agrostis, COI, ITS rRNA, Wyoming

## Abstract

In September 2020, several plants of fowl bluegrass, *Poa palustris* with seed galls were collected on a bank of river in Teton County, Wyoming, USA. Isolated nematodes were identified by both morphological and molecular methods as *Anguina agrostis.* This is a first report of *A. agrostis* in Wyoming and its report on fowl bluegrass.

The bent grass seed gall nematode, *Anguina agrostis* (Steinbuch, 1799) Filipjev, 1936 was described from *Agrostis capillaris* collected in Bavaria, Germany. Many grass species have been included in the list of host plants for this nematode. Southey (1973) and Southey et al. (1990), after reviewing literature on the host specificity of seed gall nematode populations and their morphological and morphometric differences from the type host, suggested that a thorough revision of species causing galls in the flowers of grasses was required. He proposed that *A. agrostis* appeared to be restricted to populations causing characteristic elongate galls and abnormally elongated floral structures in grasses of the genus *Agrostis*. Analysis of the ITS rRNA gene sequences made by Subbotin et al. (2004) supported the concept of a narrow specialization of seed gall nematodes and showed that *A. agrostis* was only found on *Agrostis capillaris*. Other grasses previously known as hosts for *A. agrostis* were actual hosts for several other still undescribed species of *Anguina*. *Anguina agrostis* is considered as a serious or potentially important nematode pest of bent grass in the Pacific Northwest, USA, and New Zealand (Southey, 1973; Lehman, 1979; Subbotin and Riley, 2012). In the USA, *A. agrostis* was found in Oregon, Washington (Courtney and Howell, 1952), Virginia, and Minnesota (Eisenback and Roane, 2006).

In September 2020, several plants of fowl bluegrass, *Poa palustris* L. with seed galls (6-10 mm long) (Fig. 1) were collected on a bank of river in Teton County (N 44° 35.755′, W 110° 49.866′, 7.299 feet), Wyoming, USA. Morphological and molecular analysis revealed that these galls were induced by *Anguina agrostis.* The galls were dissected in water with a binocular microscope. Only second-stage juveniles were observed from these galls. Juvenile specimens were killed by gentle heat and fixed in a solution of 4% formaldehyde for light microscopy. Measurements were made with a compound Olympus BX51 microscope equipped with Nomarski differential interference contrast. Measurements of the second-stage juveniles (*n* = 12) were: *L* = 820 ± 39 (775-905) µm; *W* = 13.5±0.8 (12.5-15.0) µm; stylet length = 8.4 ± 0.6 (6.8-8.8) µm; anterior end to median bulb valve =70±2.5 (65-75) µm; *a* = 60±3.8 (53.7-66.9); *b* = 5.5 ± 0.5 (5.0-6.5). They are similar with those provided by Southey (1973) and Chizhov (1980).

**Figure 1.**
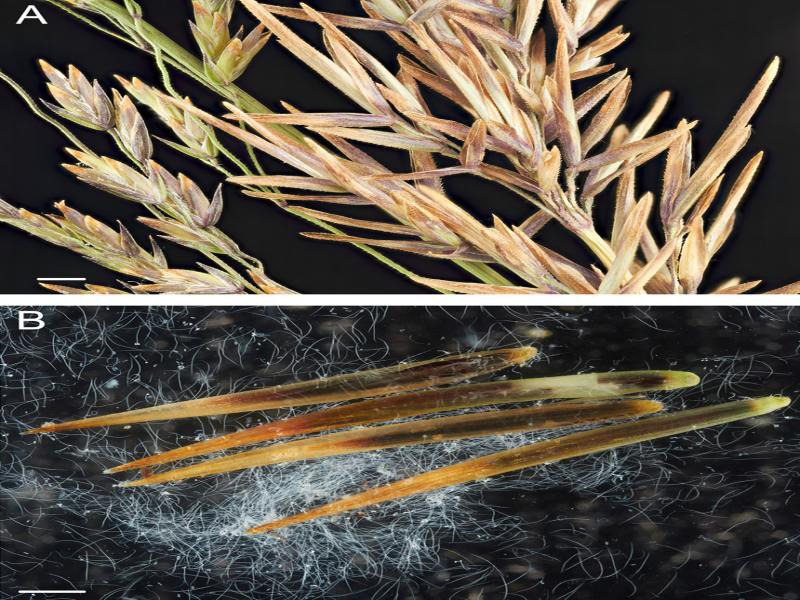
A: Inflorescence of fowl bluegrass, *Poa palustris* with normal spikelets (left) and inflorescence with nematode seed galls (right); B: Nematode seed galls with second-stage juveniles of *Anguina agrostis*. Scales – 1 mm.

DNA was extracted from several live second-stage juveniles. DNA extraction, PCR, and sequencing were performed as described by Subbotin et al. (2018). Several primer sets were used in the present study: the forward TW81 (5′-GTT TCC GTA GGT GAA CCT GC-3′) and the reverse AB28 (5′-ATA TGC TTA AGT TCA GCG GGT-3′) primers for amplification of the ITS1-5.8S-ITS2 rRNA gene and the forward AngF1-agro (5′-CGT TTA AAC TCT ATT AGT CTG TGG-3′) primer and a mixture of the reverse AnguinaR1a (5′-CCA AAA AAC CAA AAT AGA TGC TG-3′) and AnguinaR1b (5′-CCG AAG AAC CAG AAG AGG TGC TG-3′) primers. The PCR products were purified and directly sequenced in GENEWIZ (CA, USA). New sequences were deposited in the GenBank database under accession numbers: MW165338 (ITS1-5.8S-ITS2 rRNA gene) and MW174765 (*COI* gene). Blastn search showed that the ITS rRNA and *COI* gene sequences of nematode isolated from *Poa palustris* were similar in 100% (100% coverage) with corresponding genes of *A. agrostis* from the USA (MH374271 and MG321205, respectively).

Thus, morphological and molecular examination confirmed the species as *A. agrostis.* This is a first report of *A. agrostis* in Wyoming, USA and molecularly confirmed report of this species on fowl bluegrass, *Poa palustris.*
